# Prevalence and socioeconomic correlates of chronic morbidity among elderly people in Kosovo: a population-based survey

**DOI:** 10.1186/1471-2318-13-22

**Published:** 2013-03-01

**Authors:** Naim Jerliu, Ervin Toçi, Genc Burazeri, Naser Ramadani, Helmut Brand

**Affiliations:** 1 Department of International Health, School for Public Health and Primary Care (CAPHRI), Faculty of Health, Medicine and Life Sciences, Maastricht University, Maastricht, The Netherlands; 2 Institute of Public Health, Pristine, Kosovo; 3 Institute of Public Health, Tirana, Albania

**Keywords:** Aging, Chronic morbidity, Elderly, Kosovo, Multimorbidity

## Abstract

**Background:**

Our aim was to assess the prevalence and demographic and socioeconomic correlates of chronic morbidity in the elderly population of transitional Kosovo.

**Methods:**

A cross-sectional study was conducted in Kosovo in 2011 including a representative sample of 1890 individuals aged ≥65 years (949 men, mean age 73 ± 6 years; 941 women, mean age 74 ± 7 years; response rate: 83%). A structured questionnaire inquired about the presence and the number of self-reported chronic diseases among elderly people, and their access to medical care. Demographic and socioeconomic data were also collected. Binary logistic regression was used to assess the association of demographic and socioeconomic characteristics with chronic conditions.

**Results:**

In this nationwide population-based sample in Kosovo, 42% of elderly people were unable to access medical care, of whom 88% due to unaffordable costs. About 83% of the elderly people reported at least one chronic condition (63% cardiovascular diseases), and 45% had at least two chronic diseases. In multivariable-adjusted models, factors associated with the presence of chronic conditions and/or multimorbidity were female sex, older age, self-perceived poverty and the inability to access medical care.

**Conclusion:**

This study provides important evidence on the magnitude and distribution of chronic conditions among the elderly population of Kosovo. Our findings suggest that, in this sample of elderly people from Kosovo, the oldest-old (especially women) and the poor endure the vast majority of chronic conditions. These findings point to the urgent need to establish a social health insurance scheme including the marginalized segments of elderly people in this transitional country.

## Background

World population is ageing. The 1990–2009 period was characterized by an increase of life expectancy at birth in 165 out of 193 countries, with 29 countries having more than one fifth of their population aged over 60 years [1]. Conversely, in 51 nations, individuals born in 2009 are expected to live on average more than 75 years [1]. However, increased longevity does not necessarily point to good health for the extra years of life due to a positive relationship between age and disease occurrence which, coupled with the introduction of new health technologies and medical progress, will unavoidably lead to increased needs for health care services [2].

Kosovo, which emerged as the newest state of Europe in 2008 after ten years under United Nations’ administration following a devastating war [3], is trying to rebuild its health system [4]. Among the reforming efforts, an important aspect is the reorientation of health services to ensure basic medical care for all individuals and, particularly, for the vulnerable groups [3,5]. Although Kosovo’s population is the youngest in the region, it is facing the ageing phenomenon as the share of people aged ≥65 years increased from 4.6% in 1966 [6] to 6.7% in 2011 and fertility rates declined [7]. Elderly people are commonly described as a vulnerable community [8] and, in a country struggling to survive, the scientific information about health situation of elderly people is sporadic [9] and they are often overlooked in spite of their growing numbers.

Transitional countries of Southeast Europe are currently considered to be similar to developed societies in terms of morbidity and mortality patterns [5]. This may suggest changes in the epidemiological profile with an increase in the morbidity and mortality from non-communicable conditions. Indeed, the leading causes of death in Southeast European countries are now the circulatory diseases accounting for more than half of all deaths, followed by cancer [10].

Socioeconomic characteristics influence the health status of individuals. Thus, there is considerable evidence on a positive relationship between education and income with a better health status [11-13]. While numerous studies have highlighted the links between socioeconomic inequalities and health among elderly populations in developed [13-15] and developing countries [16-18], the health status of elderly people and its association with socioeconomic factors remains largely under researched in countries of the Western Balkans [19-22]. This is especially the case for chronic conditions and multimorbidity.

The magnitude and demographic and socioeconomic determinants of chronic morbidity among the elderly population in Kosovo have not been reported to date. In this context, this study aimed to assess the prevalence and the demographic and socioeconomic correlates of chronic morbidity in the elderly population of Kosovo in 2011.

## Methods

A nationwide cross-sectional study among elderly people aged ≥65 years was conducted in Kosovo, a country with an area of 10908 kilometre squares, which is divided into 37 administrative units, referred to as “communes”.

### Study population

According to the 2011 Census [7], the total population of Kosovo is 1739825 inhabitants. Our study was conducted between January and March 2011, prior to the 2011 Census’ results.

In 2010, we retrieved (from the Kosovo Ministry of Labour and Social Welfare) a list (sampling frame) containing 140329 individuals aged ≥65 years [22]. Based on this list, we drew an age- sex-and residence-stratified sample of 2400 individuals aged ≥65 years. Twelve strata were established (based on sex-stratification [men vs. women], place of residence (urban vs. rural areas) and age-stratification [65–74 years, 75–84 years and ≥85 years]). A simple random sample of 200 individuals in each of the twelve strata was drawn [22].

Of the 2400 subjects included in the sample, 135 individuals were ineligible (69 people were dead, whereas further 66 individuals had left Kosovo at the time of the survey). Of the 2265 eligible individuals, 1890 agreed to participate in the study, with an overall response rate of 83.4% (1890/2265).

### Data collection

A structured questionnaire was administered by trained interviewers at homes of elderly people who agreed to participate in the survey [22].

Participants were asked about the presence of chronic diseases (*“Do you have any long-standing or chronic illness, disease or disorder?*). Upon a positive response, participants were further asked to specify the type(s) of the following chronic conditions they were suffering from: cardio-vascular diseases (CVD), diabetes, stomach diseases, diseases of the liver, lung diseases, neurologic disorders, cancer, as well as an open-ended category about the possible presence of other chronic diseases. Based on this self-reported information, we calculated the number of chronic diseases for each participant (range: 1–6).

The questionnaire included also questions about access to medical care (*“Are you able to access medical treatment?”* – dichotomized into “able” vs. “unable” to access medical care) and barriers to access medical care (trichotomized into: “can’t afford medical costs”, “services too far away” and “too sick for seeking medical care”).

In addition, the questionnaire contained items about socio-demographic and socioeconomic characteristics of study participants including sex, age (65–74 years, 75–84 years and ≥85 years), educational level (0 years, 1–8 years and ≥9 years), place of residence (urban vs. rural area), marital status (dichotomized into: married vs. not married), and self-perceived poverty level (dichotomized into: not poor vs. poor) [22].

The study was approved by the Ethical Board of the Ministry of Health of Kosovo. All elderly people who agreed to participate in the survey signed an informed consent form prior to the interview.

### Statistical analysis

Demographic and socioeconomic sample estimates were weighted for age, sex and place of residence in accordance with the respective strata from the sampling frame [22]. Absolute numbers and their respective percentages from the study sample, and sampling frame weighted percentages with their respective 95% confidence intervals (95% CIs) were reported.

Binary logistic regression was used to assess the associations of socio-demographic and socioeconomic variables with the presence of chronic conditions (dichotomized into: “none” vs. “at least one chronic condition”) and the number of chronic conditions (dichotomized into: “one chronic condition” vs. “≥2 chronic conditions”). Individuals with ≥2 chronic conditions were considered as living with multimorbidity.

Age-adjusted and multivariable-adjusted odds ratios (ORs) and their respective 95% CIs were calculated. In all cases, a p-value of ≤0.05 was regarded as statistically significant. All the logistic models were checked to comply with the requirements of Hosmer-Lemeshow goodness-of-fit test (all the reported models satisfied the goodness-of-fit criterion).

SPSS (Statistical Package for Social Sciences, version 15.0), was used for all the statistical analyses.

## Results

Mean age in this sample of elderly people in Kosovo was 73.4 ± 6.3 years. The majority lived in rural areas (62%) and 55% were married at the time of the interview (Table 1). Almost half of participants perceived themselves as economically poor. About 42% of respondents reported inability to access medical care, most of whom (87.7%) couldn’t afford the costs of medical care.

**Table 1:** Socio-demographic and socioeconomic characteristics of a population-based sample (N = 1890) of elderly people in Kosovo, 2011

**Variable**	**Sample number (percentage)**	Weighted percentage (95% CI)^*^
**Sex:**		
Men	949 (50.2)	46.4 (46.2–46.7)
Women	941 (49.8)	53.6 (53.3–53.8)
**Age:**		
65–74 years	607 (32.1)	64.9 (64.6–65.1)
75–84 years	675 (35.7)	30.4 (30.1–30.6)
≥85 years	608 (32.2)	4.8 (4.7–4.9)
**Place of residence:**		
Rural area	973 (51.5)	62.1 (61.8–62.3)
Urban area	917 (48.5)	37.9 (37.6–38.2)
**Educational level:**		
0 years	836 (44.7)	33.7 (33.5–34.0)
1–8 years	832 (44.4)	54.1 (53.8–54.3)
≥9 years	204 (10.9)	12.2 (12.0–12.4)
**Marital status:**		
Currently married	797 (42.8)	45.3 (45.0–45.6)
Other^**†**^	1064 (57.2)	54.7 (54.4–55.0)
**Self-perceived poverty:**		
Not poor	920 (49.7)	52.2 (51.9–52.5)
Poor	930 (50.3)	47.8 (47.5–48.1)
**Access to medical care:**		
Able	1087 (57.5)	58.3 (58.1–58.5)
Unable	803 (42.5)	41.7 (41.4–41.9)
**Reasons for not accessing medical care:**^**‡**^		
Can’t afford the costs	682 (84.9)	87.7 (87.4–87.9)
Services too far away	64 (8.0)	8.0 (7.8–8.2)
Too sick for seeking care	57 (7.1)	4.3 (4.1–4.5)

^*^ Percentages and 95% confidence intervals (95% CI; in parentheses) were weighted for age-, sex- and-residence in accordance with the respective strata weights in the sampling frame.

^**†**^ Widowed, separated or divorced.

^**‡**^ Analysis restricted to individuals who were unable to access medical care (n = 803).

The most prevalent chronic conditions were the cardiovascular diseases followed by diseases of the stomach and liver, diabetes and lung diseases with 63%, 21%, 18% and 16%, respectively (Table 2). The maximum number of chronic conditions was six, whereas the median number of diseases was two. The prevalence of each chronic condition, considered separately, was higher among women than men, except for the cancer.

**Table 2:** Self-reported chronic conditions in a population-based sample (N = 1890) of elderly people in Kosovo, 2011

**Chronic condition**	**Men (N = 949)**		**Women (N = 941)**		**Total (N = 1890)**	
	n^*^	**Percentage (95% CI)**^**†**^	n^*^	**Percentage (95% CI)**^**†**^	n^*^	**Percentage (95% CI)**^**†**^
**CVD**	573	54.7 (54.3–55.1)	667	70.5 (70.2–70.8)	1240	63.2 (62.9–63.4)
**Diabetes**	121	15.0 (14.7–15.3)	170	19.8 (19.5–20.1)	291	17.6 (17.4–17.8)
**Stomach and liver**	197	18.8 (18.5–19.1)	244	23.6 (23.3–23.9)	441	21.4 (21.2–21.6)
**Lung**	170	13.7 (13.5–14.0)	181	18.4 (18.1–18.7)	351	16.2 (16.0–16.4)
**Neurologic disorders**	163	12.3 (12.1–12.6)	221	18.2 (17.9–18.5)	384	15.5 (15.3–15.7)
**Cancer**	21	1.6 (1.5–1.7)	13	1.5 (1.4–1.6)	34	1.6 (1.5–1.7)
**Other conditions**	183	14.4 (14.2–14.8)	154	15.4 (15.2–15.7)	337	15.0 (14.7–15.2)

^*^ Absolute numbers in the sample.

^**†**^ Percentages and 95% confidence intervals (95% CI; in parentheses) were weighted for age-, sex- and-residence in accordance with the respective strata weights in the sampling frame.

Women aged 65–74 years and ≥85 years were significantly more likely to report two or more chronic conditions than men (P < 0.001 and P = 0.05, respectively) [Table 3]. More than four fifths of the elderly people (83%) had at least one chronic disease, whereas the prevalence of multimorbidity (≥2 chronic conditions) was 45%.

**Table 3:** Number of chronic conditions by age and sex

**No. of chronic diseases**	**Total (N = 1890)**	**65–74 years**^**†**^		**75–84 years**^**†**^		**≥ 85 years**^**†**^	
		**(n = 607)**		**(n = 675)**		**(n = 608)**	
		**Men**	**Women**	**Men**	**Women**	**Men**	**Women**
		**(n = 307)**	**(n = 300)**	**(n = 343)**	**(n = 332)**	**(n = 299)**	**(n = 309)**
0	244 (16.7)^*^	84 (27.3)^*^	45 (14.0)^*^	42 (12.9)^*^	26 (8.2)^*^	28 (9.1)^*^	19 (7.2)^*^
1	681 (38.1)	132 (42.8)	110 (36.4)	124 (35.9)	121 (36.3)	105 (35.8)	89 (29.6)
2	605 (27.9)	60 (19.4)	86 (29.8)	113 (32.5)	111 (33.2)	108 (35.4)	127 (40.2)
≥ 3	360 (17.3)	31 (10.4)	59 (19.8)	64 (18.8)	74 (22.3)	58 (19.7)	74 (23.0)

^*^ Sample numbers and weighted percentages (in parentheses). Percentages were weighted for age-, sex- and-residence in accordance with the respective strata weights in the sampling frame.

^**†**^ Chi square test: P value <0.001 for 65–74 years, P = 0.150 for 75–84 years, and P = 0.045 for ≥85 years.

In age-adjusted logistic regression models (Table 4), sex, age-group, education, self-perceived poverty and the ability to access medical care were all significantly associated with chronic morbidity: the presence of at least one chronic condition was significantly higher in women (OR = 1.8; 95% CI = 1.4-2.4), among the oldest-old (OR = 3.2; 95% CI = 2.3-4.6), those with 1–8 years of formal schooling (OR = 2.1; 95% CI = 1.3-3.2), individuals perceiving themselves as poor (OR = 2.2; 95% CI = 1.7-3.0), and participants unable to access medical care (OR = 4.0; 95% CI = 2.8-5.7). A similar pattern was evident for multimorbidity, where OR was 1.4 times higher among women (95% CI = 1.1-1.7), 1.9 times higher among the oldest-old (95% CI = 1.5-2.5), 1.7 times higher among individuals with 1–8 years of formal schooling (95% CI = 1.2-2.4), 1.6 times higher among individuals perceiving themselves as poor (95% CI = 1.3-1.9), and 1.9 times higher among participants unable to access medical care (95% CI = 1.6-2.4) [Table 4].

**Table 4:** Association of demographic and socioeconomic factors with the presence and the number of chronic conditions; age-adjusted odds ratios (ORs) from binary logistic regression

**Variable**	Presence of chronic conditions^*^		**Multimorbidity**^**†**^	
	**OR (95% CI)**	**P**	**OR (95% CI)**	**P**
**Sex:**		<0.001		
Men	1.00 (reference)		1.00 (reference)	0.001
Women	1.85 (1.39–2.45)		1.40 (1.15–1.71)	
**Age-group:**		**<0.001 (2)**^**‡**^		**<0.001 (2)**
65–74	1.00 (reference)	-	1.00 (reference)	-
75–84	2.41 (1.75–3.31)	<0.001	1.52 (1.19–1.93)	0.001
≥85	3.22 (2.26–4.60)	<0.001	1.94 (1.51–2.49)	<0.001
**Residence:**				
Rural	1.00 (reference)	0.462	1.00 (reference)	0.866
Urban	1.11 (0.84–1.46)		1.02 (0.83–1.24)	
**Educational level:**		**0.002 (2)**		**0.005 (2)**
≥9 years	1.00 (reference)	-	1.00 (reference)	-
1–8 years	1.77 (1.21–2.59)	0.004	1.37 (0.96–1.94)	0.081
0 years	2.06 (1.35–3.16)	0.001	1.67 (1.16–2.38)	0.005
**Self-perceived poverty:**				
Not poor	1.00 (reference)	<0.001	1.00 (reference)	<0.001
Poor	2.24 (1.68–3.00)		1.56 (1.28–1.91)	
**Access to medical care:**				
Able	1.00 (reference)	<0.001	1.00 (reference)	<0.001
Unable	4.02 (2.84–5.69)		1.94 (1.58–2.37)	

^*^ OR: presence vs. absence of chronic conditions.

^**†**^ OR: multimorbidity (≥2 chronic conditions) vs. single morbidity (1 chronic condition). Individuals with no chronic conditions (n = 244) were excluded from this analysis.

^**‡**^ Overall p-values and degrees of freedom (in parentheses).

In multivariable-adjusted models (Table 5), the positive and statistically significant associations of the presence of at least one chronic condition with female sex, older age, self-perceived poverty and the inability to access medical care persisted, albeit less strongly. Furthermore, upon multivariable adjustment, the correlates of multimorbidity were generally similar to those of chronic morbidity.

**Table 5:** Association of demographic and socioeconomic factors with the presence and the number of chronic conditions; multivariable-adjusted odds ratios (ORs) from binary logistic regression

**Variable**	Presence of chronic conditions^*^		**Multimorbidity**^**†**^	
	**OR (95% CI)**	**P**	**OR (95% CI)**	**P**
**Sex:**				
Men	1.00 (reference)	0.014	1.00 (reference)	0.081
Women	1.50 (1.09–2.08)		1.23 (0.98–1.54)	
**Age-group:**		**<0.001 (2)**^**‡**^		**<0.001 (2)**
65–74	1.00 (reference)	-	1.00 (reference)	-
75–84	2.45 (1.73–3.46)	<0.001	1.46 (1.12–1.89)	0.005
≥85	2.93 (1.97–4.37)	<0.001	1.81 (1.36–2.39)	<0.001
**Residence:**				
Rural	1.00 (reference)	0.206	1.00 (reference)	0.598
Urban	1.22 (0.90–1.65)		1.06 (0.85–1.32)	
**Educational level:**		**0.150 (2)**		**0.400 (2)**
≥9 years	1.00 (reference)	-	1.00 (reference)	-
1–8 years	1.52 (0.99–2.32)	0.052	1.22 (0.84–1.77)	0.304
0 years	1.46 (0.87–2.45)	0.148	1.33 (0.88–2.03)	0.178
**Self-perceived poverty:**				
Not poor	1.00 ( reference)	0.010	1.00 (reference)	0.049
Poor	1.50 (1.10–2.05)		1.25 (1.00–1.55)	
**Access to medical care**				
Able	1.00 (reference)	<0.001	1.00 (reference)	<0.001
Unable	3.27 (2.27–4.71)		1.77 (1.41–2.20)	

^*^ OR: presence vs. absence of chronic conditions.

^**†**^ OR: multimorbidity (≥2 chronic conditions) vs. single morbidity (1 chronic condition). Individuals with no chronic conditions (n = 244) were excluded from this analysis.

^**‡**^ Overall p-values and degrees of freedom (in parentheses).

## Discussion

This study provides novel evidence about the presence and demographic and socioeconomic correlates of chronic morbidity in the elderly population of transitional Kosovo. Older age and inability to access medical care were the most consistent correlates of chronic morbidity and/or multimorbidity in this study population.

In line with other studies using similar methods for assessing chronic diseases (i.e. self-reported data) [23-26], CVD (including hypertension) was the most prevalent disease among elderly individuals in this sample. Thus, a similar prevalence of CVD has been reported among older people in the region, with a prevalence of 58% reported in Albania [21] and Serbia [20], and slightly over 50% in Macedonia [19]. Conversely, a lower prevalence varying from 28% in the Netherlands to 41% in Finland was reported by the FINE study which, however, used diagnosed rather than self-assessed measurement of chronic conditions [27].

One out of five individuals in this Kosovo sample reported diabetes, which resembles results from Albania (19%) [21], Germany (17%) [24] and USA (22.7%) [26]. Studies measuring diagnosed diabetes report a prevalence from 15% (in USA) [28] to 9% (Italy) and 6% (the Netherlands) [27]. The prevalence of cancer in our study was quite low compared to other countries in the region: 2-3% in Macedonia [19], 3% in Italy (27), about 4% in Southern Germany [24], 8% in Finland [27], and 19% among the American older people [26]. These differences could be partly explained by different methods for assessing the presence of chronic conditions (self-reported vs. diagnosed data) used in different studies, even though it has been argued that self-reports and health care records provide quite comparable estimates for diabetes, but are less concordant for chronic heart disease or other conditions [25,29]. For example, the beyond chance agreement index (Kappa statistic) has been reported at 0.90 [25] and 0.80 [29] for diabetes, 0.67 [25] and 0.40 [29] for hypertension, but lower for other chronic conditions. Methodological issues aside, a plausible reason for the discrepancies in diabetes prevalence among the elderly people between Kosovo and developed countries such as e.g. the Netherlands could be found in the epidemiology of diabetes which suggests an increasing risk with age, lower education and socioeconomic status [30,31] – factors which were all more prevalent in the Kosovo sample [22] compared with the study populations researched elsewhere [27]. Furthermore, another possible explanation for the particularly low prevalence of self-reported cancer in our study might come from a recent survey among cancer patients in Albanian settings, which found that most cancer patients seek medical help only in advanced stages of the illness and the cultural context is largely against diagnosis disclosure [32].

According to a recent systematic review [33], the prevalence of multimorbidity among the elderly, defined as the concomitant presence of ≥2 chronic conditions, ranges from 55%-98%, whereas in our study we noted a prevalence of 45%. The discrepancies might be due to different age-groups included in different studies, differences in the number of chronic conditions investigated, differences in the study settings and, as mentioned earlier, different means of assessing the presence of diseases. Furthermore, evidence shows that the prevalence of multimorbidity depends on the study population (e.g. population-based samples vs. primary care users, implying a higher prevalence in the later study population), the nature and the number of chronic conditions included [34,35]. Indeed, it has been convincingly shown that the prevalence of multimorbity increases as the number of disease items included in the questionnaire increases [34,35]. However, we tried to overcome this limitation by introducing the following option: “*Please mention any other type of chronic diseases not mentioned above*”.

Another potential source of variability between studies in estimating morbidity and multimorbidity pertains to education. As the education attainment progresses, the knowledge and understanding of health and disease changes too, leading thus to potentially different reporting. On the other hand, the awareness of people regarding health issues has been rising in general leading to more frequent doctor visits and diagnoses, especially in developed countries. Also, people now can talk more openly about their health problems and this implies greater willingness to report such problems when under study [34].

In general, the literature reports that morbidity and multimorbidity is significantly higher among older people, women and individuals of a low socioeconomic status [24,33,34,36]. Our findings are in concordance with the international literature as regards the association with sex and age, with women and the oldest-old reporting higher rates of multimorbidity.

Conversely, the inverse association with education was significant in crude analysis only. However, this resembles prior reports from studies conducted elsewhere, which have pointed out not significant relationships between education and the number of chronic conditions and multimorbidity in multivariable-adjusted models [37-40]. Thus, a study including individuals aged ≥18 years reported a non-significant association between education and multimorbidity [36], whereas a large cohort study among elderly people aged 50–75 years old reported that, upon multivariable-adjustment, the association of multimorbidity with education weakened in men, whereas in women it was not statistically significant [41].

Morbidity and multimorbidity among the elderly deserves special attention based on previous research which shows that, for certain diseases affecting the heart, lungs and circulatory apparatus, the presence of one or more chronic health conditions is significantly associated with a higher risk of death [27]. Two longitudinal studies reported that persons with poor self-reported health had an early mortality risk and late mortality risk of about three times higher compared to individuals with good health status [42,43].

About half of the elderly subjects in this study perceived themselves as poor. This might be an indicator of the difficult situation of the elderly population in Kosovo. A prior report including this very study population in Kosovo indicated that the self-perceived poverty was significantly higher among women, those without any formal schooling, urban residents and among the elderly people living alone [22].

We found significant associations of self-reported poverty with the number of chronic conditions: the higher the poverty level, the higher the proportion of multiple diseases (Spearman’s correlation coefficient = 0.212, P = 0.01; not shown in the tables). Indeed, evidence shows that even after controlling for a number of factors, poverty remains a strong predictor of adults’ health [44]. Education and poverty seem to be part of a vicious circle: low education, which is greatly influenced by unfavourable family circumstances during childhood, might be closely linked to a lower income during adulthood favouring persistent poverty which in turn contributes to poor health outcomes later in life [44]. Since the objective and subjective measures of poverty have been reported to correlate with each-other [45], self-perceived poverty might explain a part of unfavourable health outcomes among Kosovo elderly people, too. Yet, self-perceived poverty and well-being depend on many factors other than income [46].

Some of the socioeconomic and demographic determinants of chronic morbidity and multimorbidity among the elderly have been studied extensively, but little is known about other risk factors of multimorbidity including genetic, biological, lifestyle and environmental factors [33]. Another under researched factor which could affect the health status of old people is elderly abuse, which includes “abandonment, emotional abuse, financial or material exploitation, neglect, physical abuse, and sexual abuse of the elderly” [47]. Although a considerable number of studies have highlighted the situation of elderly abuse across different populations, very little evidence is available regarding the prevention of elderly abuse [47] and how this may affect the health status of older people. Elderly abuse sets an additional heavy barrier on the shoulders of older people: besides co-living with the ageing process and physical limitations that it entails, older people have to cope with the community abuse, which might further deteriorate their health status. Elderly people in Kosovo are a marginalized part of the population [22] which might imply the existence of elderly abuse. Future investigations should take into account this aspect when assessing the complexity of factors associated with morbidity of this community [36,48].

In our study, access to medical care was a significant and consistent predictor of both the presence and number of chronic conditions. The access and use of health services depends not only on the need for care, but also on predisposing characteristics (demographic factors, health beliefs) and enabling resources such as the availability of health personnel and health facilities, means of transport, or health insurance [49]. The overwhelming majority of Kosovo elderly people who couldn’t access medical care in this study (almost 90%) pointed to the economic barriers as the main reason for this inability. This is a reflection of the unclear situation of the elderly in Kosovo and the ongoing reforms in the health sector. Although protection of the rights of vulnerable groups and ensuring quality of care is one of the priorities of health reforms in Kosovo, the health system lags behind its optimal state. The health insurance system seems unable to function with half of the population unemployed and a high informality rate [3,50]. People aged ≥65 years in Kosovo rely on the social security pension (which is quite low and not sufficient to meet their everyday needs) and remittances from their close family working abroad [22]. Furthermore, Kosovo is in urgent need of deep reforms as the armed conflict left the country with a very inefficient health system characterized by a lack of trained personnel and disparities in health force distribution. These factors lead to variations in access to primary care, corruption and informal payments, which are all reflected in unfavourable child and adult health indicators. In this context, the continuous reforming of the health sector has brought up a complex configuration of the stakeholders operating in the health system which contributes to unequal access to health care. The primary health care is still overlooked by health policies which often favour “high-tech” clinical medicine [3]. Furthermore, the private health sector has been expanded rapidly, but private facilities are unaffordable for the elderly [3]. The main barrier to access care is the cost of services, despite the fact that basic health services are supposed to be covered for all citizens. Under-the-table payments put a heavy burden on the shoulders of the poor. Ultimately, the reforms have resulted in lower access to health care for the poorer groups of the society [50]. Under these conditions, little attention is paid to the growing community of the elderly people in Kosovo [22] which, combined with the inadequacy of financial resources, the economic insecurity and the unclear and unstable development of the health sector, pose a serious barrier for elderly people to access medical care.

As stated by the Centre on Social Disparities in Health [51], in order to increase the chances of good health one needs to adopt a healthy lifestyle and have access to proper medical care. In a broader context, there is a need to promote a healthier living and working conditions. This should be supported by economic development, reducing poverty and enhancing education [51].

Our study has several limitations including its cross-sectional design and the differential reporting of chronic diseases among elderly people. We cannot exclude the possibility of reporting bias; however, we do not have sound reasons to assume differential reporting of chronic diseases for the elderly people’s categories differing in demographic and socioeconomic characteristics. More importantly, findings of our study should be interpreted with caution, since the observed associations from cross-sectional studies are not assumed to be causal.

## Conclusions

Elderly people represent a valuable part of the society as they convey their wisdom and experience to future generations. Improving their economic situation and health status in Kosovo will require a lot of efforts. Although access to medical care is not the only element in the wide array of health determinants, based on our findings, medical care plays an important role for the control of chronic morbidity and multimorbidity. Therefore, facilitating the access to medical care of the elderly people in Kosovo through economic development, poverty reduction and the establishment of an effective social health insurance system might improve the health status of older people and protect them from catastrophic health expenditures.

In conclusion, our study provides evidence on the magnitude and demographic and socioeconomic correlates of chronic conditions among the elderly population of Kosovo. Our findings suggest that the oldest-old (especially women) and the poor segments of the elderly population endure the vast majority of chronic conditions. These salient findings point to the need for establishing an effective social health insurance scheme including the marginalized subgroups of elderly people in Kosovo.

## Competing interests

The authors declare that they have no competing interests.

## Authors’ contributions

NJ contributed to the study conceptualization and design, acquisition of the data, analysis and interpretation of the data and writing of the article. ET, GB and HB contributed to the study conceptualization and design, analysis and interpretation of the data and writing of the article. NR commented on the manuscript. All authors have read and approved the submitted manuscript.

## Pre-publication history

The pre-publication history for this paper can be accessed here:

http://www.biomedcentral.com/1471-2318/13/22/prepub
